# Enhanced NF-κB activation via HIV-1 Tat-TRAF6 cross-talk

**DOI:** 10.1126/sciadv.adi4162

**Published:** 2024-01-19

**Authors:** Yang Li, Xi Liu, Koh Fujinaga, John D. Gross, Alan D. Frankel

**Affiliations:** ^1^ Department of Biochemistry and Biophysics, University of California, San Francisco, CA 94158, USA.; ^2^ Department of Pharmaceutical Chemistry, University of California, San Francisco, CA 94158, USA.; ^3^ Department of Medicine, University of California, San Francisco, CA 94143, USA.

## Abstract

The Tat proteins of HIV-1 and simian immunodeficiency virus (SIV) are essential for activating viral transcription. In addition, Tat stimulates nuclear factor κB (NF-κB) signaling pathways to regulate viral gene expression although its molecular mechanism is unclear. Here, we report that Tat directly activates NF-κB through the interaction with TRAF6, which is an essential upstream signaling molecule of the canonical NF-κB pathway. This interaction increases TRAF6 oligomerization and auto-ubiquitination, as well as the synthesis of K63-linked polyubiquitin chains to further activate the NF-κB pathway and HIV-1 transcription. Moreover, ectopic expression of TRAF6 significantly activates HIV-1 transcription, whereas TRAF6 knockdown inhibits transcription. Furthermore, Tat-mediated activation of NF-κB through TRAF6 is conserved among HIV-1, HIV-2, and SIV isolates. Our study uncovers yet another mechanism by which HIV-1 subverts host transcriptional pathways to enhance its own transcription.

## Teaser

The interaction between HIV-1 Tat and TRAF6 subverts the host transcriptional machinery to activate NF-κB.

## INTRODUCTION

Nuclear factor κB (NF-κB) is a master regulator of immune signaling pathways and plays complex roles during the replication of primate lentiviruses. It is constitutively activated in HIV-1–infected primary monocytes and chronically infected myeloid cell lines (*1*–*6*). NF-κB plays significant roles in cellular antiviral responses (*7*). However, primate lentiviruses have evolved mechanisms to combat innate immune responses first by boosting NF-κB activation to initiate viral transcription at early phases of infection and suppressing it at late phases to escape the immune response (*1*–*3*). HIV-1 encodes a variety of proteins using different open reading frames and splicing mechanisms to subvert host functions and generate infectious virus particles (*8*, *9*). At the early phase of HIV-1 infection, several viral proteins (Tat, Vpr, Nef, and gp41) have been reported to enhance NF-κB activation to promote viral gene transcription, while at the late phase, other viral proteins (e.g., Vpu and Vpr) inhibit NF-κB activation to minimize antiviral gene expression (*10*–*12*).

Tat is a small protein of 86 to 101 amino acids (depending on the virus subtype) and is encoded by two exons within the viral genome. It is largely responsible for enhancing viral transcription by hijacking host transcription elongation factor P-TEFb (*13*–*17*). It is also well established that the basal level of HIV-1 transcription can be stimulated by activation of the canonical NF-κB pathway (*18*, *19*). NF-κB binding sites are found at the promoter-proximal (enhancer) region of all primate lentiviral long terminal repeats (LTRs), although the number of sites varies among different HIV-1 subtypes (*20*–*23*). Earlier studies showed that Tat can enhance NF-κB activity through different host factors, such as IκBα, CBP/p300, the double-stranded RNA-dependent protein kinase (PKR), and protein kinase C (PKC) (*24*–*28*). However, it is unclear whether other mechanisms could also be involved.

Our previous studies identified UBE2O, an E2/E3 hybrid protein, as a Tat-interacting factor involved in activating HIV-1 transcription (*29*, *30*). Other studies also found UBE2O as an inhibitor of NF-κB activation, mediated by interactions with tumor necrosis factor receptor–associated factor 6 (TRAF6) (*31*). TRAF6 is a RING E3 ligase and plays a vital role in the canonical NF-κB pathway. The oligomerization of TRAF6 can promote auto-ubiquitination and catalyzes the synthesis of K63-linked polyubiquitin chains, which can be conjugated to TRAF6 itself or exist as free ubiquitin chains (*32*–*34*). Both ubiquitinated-TRAF6 and free K63-linked polyubiquitin chains can further activate the downstream NF-κB pathway (*33*–*36*). TRAF6-mediated activation occurs via phosphorylation of the IκB kinase (IKK) complex by TAK1 (*33*, *37*, *38*). The knockdown of TRAF6 inhibits HIV-1 infection, suggesting that it is a host dependency factor (*39*); moreover, TRAF6 mRNA increases in HIV-1–infected MT2 cells (*40*). While these observations are provocative, the mechanism of how TRAF6 promotes HIV-1 infection is unclear.

In this study, we first found that although UBE2O is a Tat-interacting factor, the interaction between Tat and UBE2O is dispensable for NF-κB activation. Instead, Tat can enhance NF-κB activation through direct interaction with TRAF6 in an E3 ligase–dependent manner. TRAF6-mediated NF-κB activation by Tat is independent of P-TEFb binding and thus distinct from the role of Tat in enhancing HIV-1 elongation. We also identified several key residues in the cysteine-rich domain and core region of Tat that are critical for TRAF6-mediated NF-κB activation. These residues are highly conserved among SIV and HIV isolates, and various SIV Tat proteins also enhance TRAF6-dependent NF-κB activation. These studies highlight a new mechanism for enhancing HIV-1 transcription through TRAF6 and further demonstrate the multifunctional roles of Tat in virus transcription.

## RESULTS

### Tat enhances TRAF6-dependent NF-κB activation in a UBE2O-independent manner

**Fig. 1.**
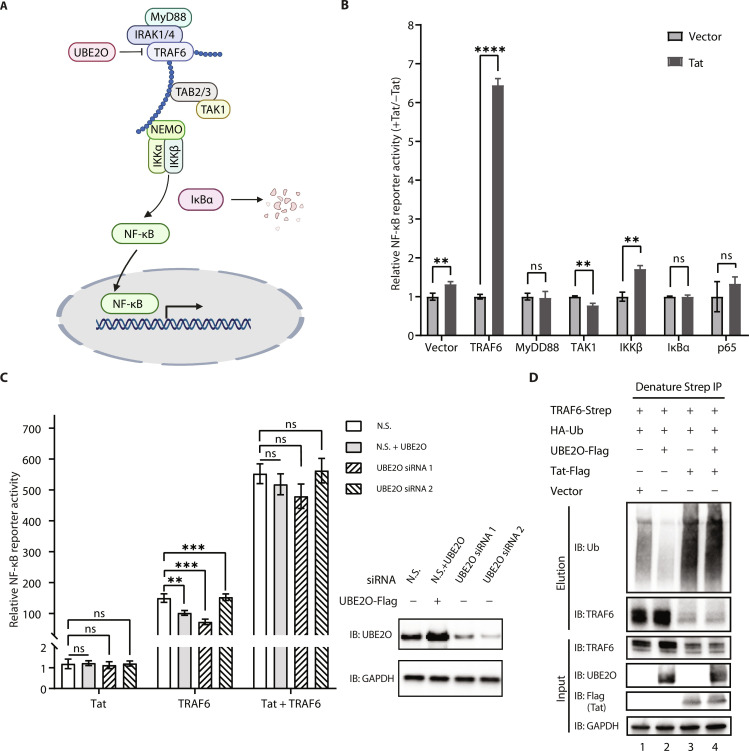
Tat enhances TRAF6-dependent NF-κB activation in a UBE2O-independent manner. (**A**) Schematic of TRAF6-mediated canonical NF-κB activation. (**B**) Identification of cellular signaling molecules related to NF-κB activation by HIV-1 Tat. Human embryonic kidney (HEK) 293T cells were transfected with an NF-κB luciferase plasmid reporter and the internal control NanoLuc luciferase (NLuc) as well as expression plasmid encoding each of the candidate proteins involved in the NF-κB signaling (MyD88, TRAF6, TAK1, IKKβ, IκBα, and p65), together with an empty vector or the plasmid encoding Tat for 24 hours. NF-κB activation was analyzed by using the Nano-Glo Dual-Luciferase Reporter Assay. (**C**) Left: NF-κB reporter luciferase activity was measured in HEK293T cells previously transfected for 48 hours with small interfering RNA (siRNA) against UBE2O (siUBE2O 1 or siUBE2O 2) or non-silencing (N.S.) siRNA and then cotransfected for 24 hours with an empty vector, Tat, TRAF6, or TRAF6 + Tat together with an NF-κB luciferase reporter and the internal control NLuc. Right: Western blot analysis of UBE2O protein expression. (**D**) In vivo ubiquitination of ectopically expressed Strep-tagged TRAF6 and hemagglutinin (HA)-ubiquitin in the presence or absence of Tat-Flag, UBE2O-Flag, or Tat-Flag/UBE2O-Flag. Cells were lysed under denaturing conditions, and IPs were performed with Strep-Tactin resin to detect ubiquitination by Western blot with an anti-ubiquitin antibody. The graphs in (B) show the means ± SD of three biological replicates and were normalized to cotransfected NLuc activities. The graphs in (C) show the means ± SD of four biological replicates and were normalized to cotransfected NLuc activities. ns *P* > 0.05, *P < 0.05, **P < 0.01, ***P < 0.001, and ****P < 0.0001, and statistical significance was assessed by a two-tailed unpaired Student’s *t* test.

To determine the molecular mechanisms by which Tat potentiates canonical NF-κB signaling, HIV-1 Tat and each candidate protein involved in NF-κB signaling (MyD88, TRAF6, TAK1, IKKβ, IκBα, and p65; Fig. 1A) were coexpressed in human embryonic kidney (HEK) 293T cells, together with an NF-κB luciferase reporter containing five copies of an NF-κB response element located upstream of firefly luciferase and NanoLuc luciferase (NLuc) as a normalization control. We found that the activation of NF-κB by TRAF6 was markedly enhanced by Tat, in contrast to the other factors, suggesting that Tat potentiates the NF-κB pathway upstream of TAK1, downstream of MyD88, most likely by targeting TRAF6 (Fig. 1B and fig. S1A). Tat enhanced TRAF6-mediated NF-κB activation in a dose-dependent manner (fig. S1B).

Since UBE2O is a Tat-interacting factor and an inhibitor of TRAF6 (Fig. 1A), we wished to know whether the effect on TRAF6-mediated NF-κB activation was mediated through UBE2O but found no obvious effect in UBE2O knockdown cells (Fig. 1C). Since UBE2O negatively regulates TRAF6-mediated NF-κB activation by inhibiting TRAF6 polyubiquitination (Fig. 1D, lanes 1 and 2), we also tested whether Tat affected ubiquitination of TRAF6. Tat significantly enhanced the ubiquitination of TRAF6 (Fig. 1D, lanes 1 and 3). Overexpression of UBE2O failed to block Tat-mediated TRAF6 ubiquitination (Fig. 1D, lanes 3 and 4). Together, these results suggest that Tat activates TRAF6-mediated NF-κB expression in a UBE2O-independent manner.

To determine the specificity between TRAF6 and Tat, we next tested whether other HIV-1 regulatory and accessory proteins can enhance tumor necrosis factor–α (TNF-α)–induced NF-κB signaling. We stimulated HEK293T cell with TNF-α after transfection with plasmids encoding each HIV-1 protein (Tat, Rev, Nef, Vif, Vpr, and Vpu), as well as an NF-κB luciferase reporter and the internal control NLuc. Notably, the viral proteins had different effects on NF-κB activation under TNF-α stimulation (fig. S1C). Tat potentiated the NF-κB activity by ~2-fold. Vif also stimulated the NF-κB activity to a lesser extent, possibly because of Vif’s lower expression level. Rev and Nef had no significant effect, whereas Vpr and Vpu exhibited slight inhibition of NF-κB. The effects of Vpr are controversial as both stimulatory and inhibitory effects on NF-κB have been reported (*10*, *41*–*43*). Vpu, as a late viral protein, potently suppresses NF-κB activation during later stages of the viral replication cycle (*11*), which is consistent with our observation. Next, a series of NF-κB luciferase assays were performed by cotransfecting TRAF6 and each of these HIV-1 proteins. Among the tested viral proteins, only Tat markedly enhanced NF-κB activation when TRAF6 was coexpressed (fig. S1D), further demonstrating high specificity.

To further test whether endogenous TRAF6 was involved in activation of the NF-κB pathway by Tat, we measured endogenous IκBα levels in the presence or absence of Tat or TRAF6, as IκBα is an inhibitory marker downstream of canonical NF-κB pathway (Fig. 1A). As expected, IκBα decreased when Tat was expressed (fig. S1E, compare lane 2 to lane 1), while the level of IκBα did not change when TRAF6 was knocked down by small interfering RNA (siRNA) (fig. S1E, compare lane 4 to lane 3), indicating that NF-κB activation by Tat is upstream of IκBα.

### Tat enhances NF-κB activation through direct interaction with TRAF6

**Fig. 2.**
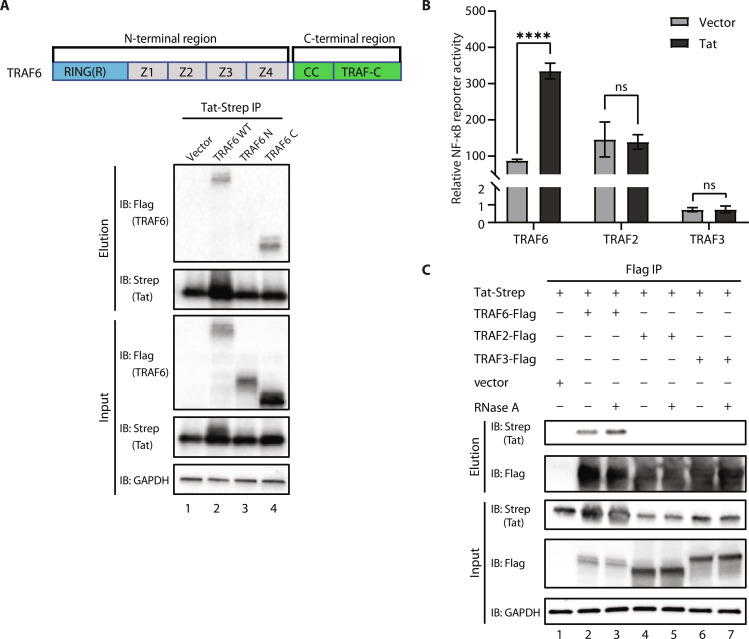
Tat enhances NF-κB activation through direct interaction with TRAF6. (**A**) Top: Domain representation of TRAF6. Bottom: Flag-tagged wild-type TRAF6 and truncation mutants (TRAF6 N or TRAF6 C) were cotransfected with Tat-Strep (Tat-S) into HEK293T cells. Tat was immunoprecipitated using an anti-Strep antibody to assess the interaction with TRAF6. (**B**) Luciferase reporter assays to measure NF-κB activation in HEK293T cells transfected with empty vector or plasmids encoding TRAF6, TRAF2, or TRAF3 together with an empty vector or Tat for 24 hours. (**C**) Co-IP analysis (with anti-Flag) and Western blot analysis (with anti-Flag or anti-Strep) of HEK293T cells transfected with plasmids encoding Tat-Strep and Flag-tagged TRAF6, TRAF2, TRAF3, or the empty vector for 48 hours. The graphs in (B) show the means ± SD of three biological replicates and were normalized by cotransfected NLuc activities. ns *P* > 0.05, ****P < 0.0001, and statistical significance was assessed by a two-tailed unpaired Student’s *t* test. GAPDH, glyceraldehyde-3-phosphate dehydrogenase.

Next, we sought to determine whether there is a direct interaction between TRAF6 and Tat. Co-immunoprecipitation (co-IP) experiments revealed that Tat can interact with TRAF6 (Fig. 2, A and C). As a multidomain protein, TRAF6 can be generally divided into N-terminal and C-terminal regions. The N-terminal region contains a RING domain followed by several zinc finger domains, and the C-terminal region has a coiled-coil domain and a unique TRAFC domain (Fig. 2A). Co-IP experiments demonstrated that the C-terminal region of TRAF6, but not the N-terminal region, is required for the interaction with Tat (Fig. 2A). There are seven TRAF proteins in the human genome and six of them contain a very similar C-terminal TRAF domain (*44*). The TRAF domains of TRAF2, TRAF3, and TRAF6 share more than 20% sequence similarity with very similar folds and have been reported to interact with various intracellular signaling molecules (*45*, *46*). It is known that ectopic expression of TRAF2 and TRAF6 enhances NF-κB activation (*47*), whereas ectopic expression of TRAF3, as an inhibitor of NF-κB pathway (*48*), shows no activation of NF-κB. We tested whether the enhancement of NF-κB activation by Tat is specific to TRAF6 by NF-κB reporter luciferase assays. Tat stimulated TRAF6-mediated activation of NF-κB more than fourfold but showed no effect on TRAF2 or TRAF3 (Fig. 2B). Despite the similarities between their TRAF domains, only TRAF6 interacted with Tat in co-IP experiments (Fig. 2C, compare lane 2 to lanes 4 and 6), and the interaction is independent of RNA (Fig. 2C, compare lane 2 to lane 3).

To further map Tat-TRAF6 interacting regions, we performed in vitro pull-down assays with various truncations of Tat fused to GST and TRAF6 C-terminal truncations fused to maltose-binding protein (MBP). Tat 1-48 and 1-72 interacted with the TRAF6 C-terminal with or without its coiled-coil domain, while Tat 1-20 did not (fig. S2A). Reciprocal pull-downs using MBP-fused TRAF6 as preys gave similar results, where regardless of the coiled-coil domain, TRAF6 interacted with Tat 1-48 and 1-72 but not Tat 1-20 (fig. S2B). On the basis of band intensities, the pull-down assays suggested the interaction between TRAF6 and Tat is weak. To test whether a tighter Tat-TRAF6 interaction would potentiate NF-κB activation, we constructed a single-chain Tat-TRAF6 fusion protein (TRAF6/Tat) and found that it did stimulate higher NF-κB activation despite its lower expression level (fig. S2C). Removing the C terminus of Tat from the fusion protein (TRAF/Tat 1-20) completely abolished the enhancement of NF-κB luciferase activity, consistent with the pull-down results, which indicates that a physical interaction between Tat and TRAF6 is required for the enhancement of NF-κB activity (fig. S2D, bar 3). TRAF6/Tat 1-58 has a lower activity, likely due to its lower expression level or protein instability (fig. S2D, bar 6). The single-chain construct with Tat 20-40 showed similar NF-κB activation as full-length Tat, indicating that the cysteine-rich region of Tat is the minimal region to enhance TRAF6-mediated NF-κB activation (fig. S2D, bar 8).

### Tat enhancement of NF-κB activation is TRAF6 E3 ligase–dependent

**Fig. 3.**
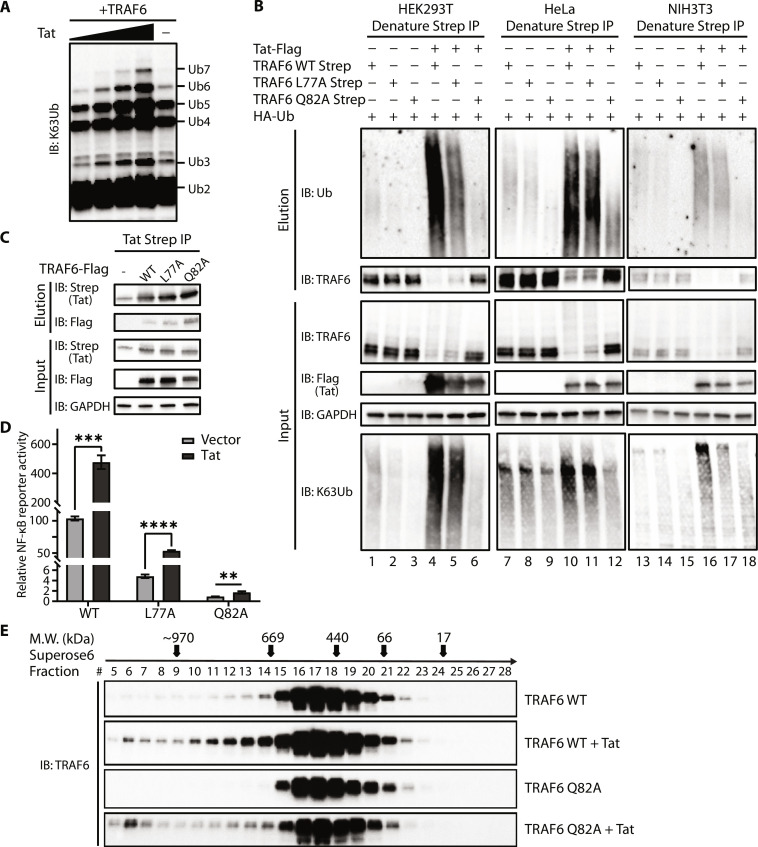
Enhancement of NF-κB activation by Tat is TRAF6 E3 ligase dependent. (**A**) Tat promotes free K63-linked polyubiquitin chain formation in vitro. In vitro ubiquitination assays were performed in the presence of E1, UBE2N/Uev1a, TRAF6, and ubiquitin with or without Tat. Samples were analyzed by Western blotting using the anti-ubiquitin (K63 linkage–specific) antibody. (**B**) In vivo ubiquitination of wild-type or mutant TRAF6 in HEK293T, HeLa, or NIH3T3 cells cotransfected with HA-ubiquitin together with an empty vector or Tat-encoding plasmid. Cells were lysed under denaturing conditions, and IPs were performed with Strep-Tactin resin to detect ubiquitination by Western blot with an anti-ubiquitin antibody. (**C**) Co-IP analysis and Western blot analysis (with anti-Flag or anti-Strep) of HEK293T cells transfected with plasmids encoding Strep-tagged Tat and Flag-tagged TRAF6, or TRAF6 mutants (L77A and Q82A) for 48 hours. (**D**) Luciferase reporter assays to measure NF-κB activation in HEK293T cells transfected with the empty vector or Tat-encoding plasmid together with plasmids encoding wild-type or mutant TRAF6 for 24 hours. Data are represented as means ± SD of three biological replicates and were normalized to cotransfected NLuc activities. **P < 0.01, ***P < 0.001, ****P < 0.0001, and statistical significance was assessed by a two-tailed unpaired Student’s *t* test. (**E**) Whole-cell lysates of HEK293T cells transfected with either wild-type TRAF6 or TRAF6 Q82A with or without Tat for 48 hours were prepared and separated on a Superose 6 Increase 10/300 GL gel filtration column. Fractions were collected and subjected to SDS–polyacrylamide gel electrophoresis and probed with anti-TRAF6 antibody. Fraction numbers are indicated at the top of the gel. Molecular mass markers were run under the same conditions and are shown on top with arrows corresponding to the fraction that represents the peak of their elution profile.

To explore the mechanism by which Tat enhances TRAF6-mediated NF-κB activation, we performed in vitro ubiquitination assays using purified recombinant TRAF6 in the presence or absence of Tat. Free K63-linked polyubiquitin chains are known to activate NF-κB signaling (*49*). We observed that Tat increased free K63-linked polyubiquitin chain production in a dose-dependent manner using an anti–K63 linkage–specific antibody (Fig. 3A and fig. S3A). Tat enhanced auto-ubiquitination of TRAF6 in vivo in three different cell lines (HEK293T, HeLa, and NIH3T3) (Fig. 3B, lanes 4, 10, and 16). In vivo pull-down assays using recombinant GST-USP5 (ZnF-UBP), which binds free ubiquitin chain diglycine carboxyl tails, also indicated Tat-enhanced free K63-linked polyubiquitin chain production (fig. S3B, compare lane 4 to lane 2) (*50*). To test the importance of TRAF6 E3 ligase activity, we examined the TRAF6 mutants L77A and Q82A which partially or completely abolish E3 ligase activities, respectively (*51*). These mutations did not reduce the interaction between Tat and TRAF6 (Fig. 3C). However, L77A showed lower ubiquitination and enhancement in the presence of Tat, whereas Q82A shows barely any ubiquitination or enhancement by Tat (Fig. 3B, lanes 5, 6, 11, 12, 17, and 18; and fig. S3B, lane 5). Robust K63-linked ubiquitination was observed only when Tat was cotransfected with wild-type TRAF6 (Fig. 3B, input with anti-K63Ub). The ability of Tat to enhance NF-κB activation was markedly reduced with these mutants, indicating that Tat stimulation requires E3 ligase activity of TRAF6 (Fig. 3D). Together, these data demonstrate that Tat enhances the synthesis of K63-linked polyubiquitin chains in vitro as well as the auto-ubiquitination of TRAF6 in vivo.

Since the E3 ligase activity of TRAF6 is associated with TRAF6 oligomerization (*52*, *53*), we examined TRAF6 oligomerization in the presence or absence of Tat by size exclusion chromatography. Both wild-type TRAF6 and Q82A appeared in significant amounts in fractions 14 through 21 (Fig. 3E), which is consistent with previous findings (*54*). In cells coexpressing TRAF6 and Tat, we observed populations of both wild-type and Q82A in earlier fractions (fractions 5 to 8), suggesting higher molecular weight forms of TRAF6 (>669 kDa) in the presence of Tat. Our results suggest that Tat regulates TRAF6 auto-ubiquitination and production of free K63-linked polyubiquitin chains via enhanced oligomerization.

### TRAF6 mediates Tat-dependent HIV-1 transcription

**Fig. 4.**
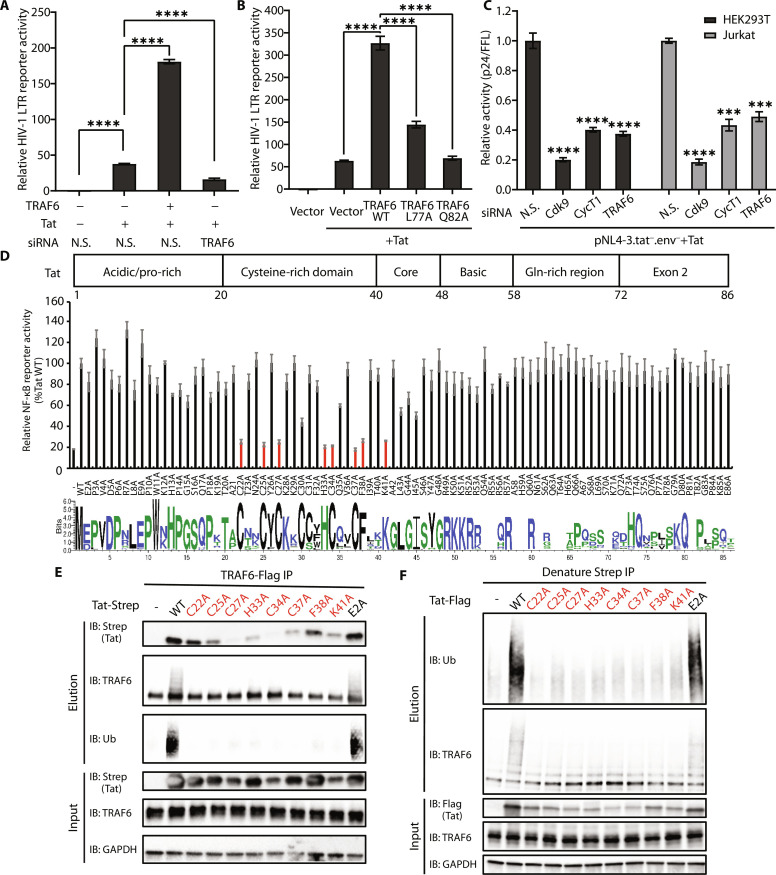
TRAF6 is a critical host factor of Tat transcriptional activity. (**A**) Relative HIV-1 LTR transcriptional activities of Tat were measured in HEK293T cells transfected for 48 hours with siRNA against TRAF6 or N.S., followed by transfection with an empty vector, Tat, TRAF6, or TRAF6 + Tat together with HIV LTR–driven firefly luciferase reporter and the internal control NLuc for 24 hours. (**B**) Relative HIV-1 LTR transcriptional activities of Tat after cotransfection with wild-type or mutant TRAF6. (**C**) Relative Tat activation of p24 expression normalized to firefly luciferase activities after siRNA knockdown of CycT1, Cdk9, and TRAF6. (**D**) Top: Domain organization of HIV-1 Tat (HXB2). Middle: Luciferase reporter assays to measure NF-κB activities in HEK293T cells transfected with TRAF6 and wild-type or mutant Tat for 24 hours. Bottom: Sequence logos showing conservation of HIV-1 Tat sequences from the Los Alamos HIV database (www.hiv.lanl.gov) (*77*). The figure was generated by WebLogo 3 (*78*). (**E**) Co-IP analysis (with anti-Flag) and Western blot analysis of HEK293 cells transiently transfected with Flag-tagged TRAF6, Strep-tagged wild-type, or mutant Tat proteins. Mutations that affect NF-κB activation are labeled in red. (**F**) In vivo ubiquitination of TRAF6 in HEK293T cells cotransfected with HA-ubiquitin together with an empty vector or Flag-tagged wild-type or mutant Tat plasmid. Cells were lysed under denaturing conditions. Data are represented in (A) as means ± SD of three biological replicates, and the graphs in (B) and (D) show the means ± SD of four biological replicates and were normalized to cotransfected NLuc luciferase activities. Data are represented in (C) as the means ± SD of three biological replicates for each siRNA transfection. ns *P* > 0.05, *P < 0.05, **P < 0.01, ***P < 0.001, ****P < 0.0001, and statistical significance was assessed by a two-tailed unpaired Student’s *t* test. The statistical significance of (D) is in the data file S1.

The HIV-1 LTR contains two NF-κB sites, so we wished to explore the possible impacts of TRAF6 on HIV-1 transcription. We first investigated the effects of endogenous or ectopically expressed TRAF6 in LTR-driven firefly luciferase (FLuc) assays. Depletion of endogenous TRAF6 by siRNA decreased HIV transcription in the presence of Tat by more than twofold compared to non-silencing (N.S.) siRNA (Fig. 4A, bars 2 and 4). Conversely, HIV transcription was enhanced by more than fourfold when TRAF6 was coexpressed with Tat (Fig. 4A, bars 2 and 3). Overexpression of TRAF6 L77A or Q82A loss-of-function mutants weakened or abolished the enhancement (Fig. 4B), consistent with previous data indicating a requirement of TRAF6 E3 ligase activity for enhancing NF-κB signaling.

To compare the effect of TRAF6 to other well-studied HIV-1 transcription factors, we used siRNA to the knockdown of TRAF6, Cdk9, and CycT1 in HEK293T and Jurkat cells and measured p24 production after transfecting the HIV-1 proviral clone pNL4-3.tat^−^.env^−^ (*55*), Tat plasmid, and the internal control pCMV-FFL (firefly luciferase). As expected, the knockdown of Cdk9 or CycT1 significantly reduced p24 levels (Fig. 4C, bars 2, 3, 6, and 7; and fig. S4, A and B). A similar effect was also seen with TRAF6 knockdown (Fig. 4C, bars 4 and 8; and fig. S4, A and B). The knockdown of TRAF6 in Jurkat cells also reduced p24 production after infection with the NL4-3 (Tat-SF) virus (fig. S4C). In the infection experiments, the virally encoded Tat also interacted with endogenous TRAF6, enhanced K63-linked ubiquitination of TRAF6, and decreased IκBα levels (fig. S4D). We also examined the effect of endogenous TRAF6 on HIV-1 reactivation from latency. TRAF6 was knocked down by siRNA in J-Lat6.3 cells, which harbors a latent HIV-derived green fluorescent protein (GFP) reporter (*56*). The cells were then stimulated with TNF-α or phorbol 12-myristate 13-acetate (PMA) to reactivate HIV, which was quantified by fluorescence-activated cell sorting (FACS) analysis. TRAF6 knockdown resulted in a significant decrease of GFP-positive cells after stimulation (fig. S4E). Together, these results suggest that TRAF6 is a novel host factor for Tat in HIV-1 transcription and for latency reactivation through the regulation of NF-κB activity.

We next screened single-point mutants of Tat to identify residues important for TRAF6-mediated NF-κB activation. Mutation of five cysteines (Cys22, Cys25, Cys27, Cys34, and Cys37) and His33, which are involved in zinc coordination (*57*), significantly reduced NF-κB activation by Tat (Fig. 4D and fig. S4F). Lys41, which forms intramolecular hydrogen bonds within the structured Tat core (*57*), was similarly important for NF-κB activation (Fig. 4D and fig. S4F). Co-IP experiments indicated that the interaction between Tat and TRAF6 was weakened upon mutation of these residues (C22A, C25A, C27A, H33A, C34A, C37A, or K41A) (Fig. 4E). Furthermore, these Tat mutants were incapable of inducing TRAF6 ubiquitination (Western blot against ubiquitin in elution; Fig. 4, E and F). Earlier work has shown that Phe38, a relatively exposed residue in Tat (fig. S4F), plays a critical role in HIV-1 transcription (*58*). Tat F38A completely failed to trigger the auto-ubiquitination of TRAF6 and decreased the production of free K63-linked polyubiquitin chains, while it exhibited slightly weakened interaction with TRAF6 (Western blot against ubiquitin in elution; Fig. 4, E and F, and fig. S3B, compare lane 6 to lane 4). In addition, these mutants exhibited no significant difference in NF-κB activation without overexpression of TRAF6 (fig. S4G). These results indicate that the cysteine-rich region and Phe38 are involved in the formation of the binding surface for TRAF6.

### TRAF6-mediated NF-κB activation by Tat is independent of P-TEFb binding

**Fig. 5.**
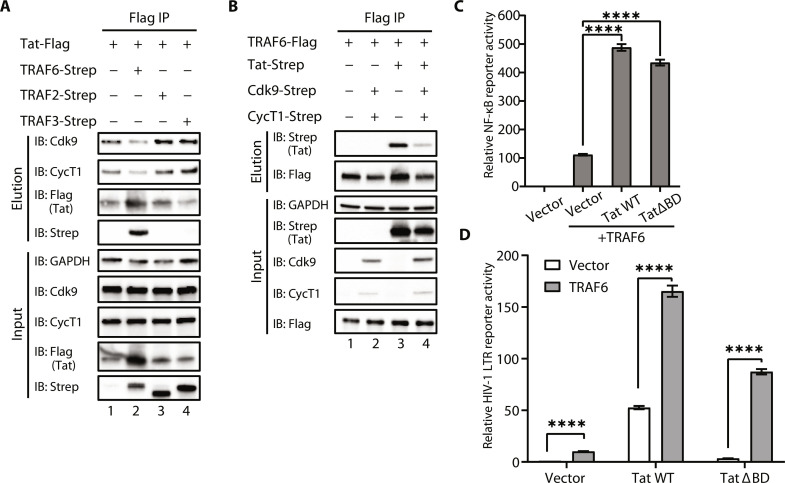
TRAF6-mediated NF-κB activation by Tat is independent of P-TEFb binding. (**A**) The association of CycT1 or Cdk9 and HIV-1 Tat upon transient transfection with TRAF6, TRAF2, TRAF3, or vector in HEK293T cells was analyzed by co-IP assays. (**B**) The association of TRAF6 and HIV-1 Tat upon transient transfection with CycT1 and Cdk9 in HEK293T cells was analyzed by co-IP assays. (**C**) Luciferase reporter assays to measure NF-κB activity in HEK293T cells transfected with TRAF6 and wild-type or ∆BD Tat for 24 hours. (**D**) Relative HIV-1 LTR transcriptional activity of wild-type or ∆BD Tat after cotransfection with TRAF6 or an empty vector. Data in (C) and (D) are represented as means ± SD of four biological replicates and were normalized to cotransfected NLuc luciferase activities. ****P < 0.0001, and statistical significance was assessed by a two-tailed unpaired Student’s *t* test.

The cysteine-rich region of Tat is involved in both P-TEFb and TRAF6 binding, so we sought to determine whether these interactions are mutually exclusive. Co-IP assays indicated that overexpression of TRAF6, but not TRAF2 or TRAF3, decreased the association of Tat with CycT1 and Cdk9 (Fig. 5A). Conversely, overexpression of CycT1 and Cdk9 decreased Tat-TRAF6 binding (Fig. 5B, lanes 3 and 4). Together, our results suggest that the binding of Tat to TRAF6 is mutually exclusive with P-TEFb.

To activate viral transcription elongation, the Tat-P-TEFb complex binds to the TAR RNA element positioned at the 5′ end of the viral transcripts, in part via the basic domain of Tat (*59*–*61*). Previous studies demonstrated that Nullbasic Tat, which has the basic domain replaced with glycine/alanine residues, is mainly localized in the cytoplasm and still interacts with P-TEFb, but it is unable to bring the ternary complex to TAR in the nucleus (*62*). Consequently, it inhibits Tat activation and virus replication (*15*, *63*). As expected, Tat ∆BD in which the basic domain was deleted, also localized predominantly in the cytoplasm (fig. S5A). However, ∆BD maintained the ability to enhance TRAF6-dependent NF-κB activation (Fig. 5C). TRAF6 also increased HIV-1 transcriptional activity in the presence of ∆BD but significantly lower compared to wild-type Tat (Fig. 5D). These data suggest that the TRAF6-dependent NF-κB enhancement by Tat primarily takes place within the cytoplasm.

Structure-based alignments of TRAF6 complexes with different peptides demonstrated that F471 and Y473 in the TRAF6’s C-terminal domain form a hydrophobic pocket that interacts with proline in receptor peptides (*64*). Therefore, we tested whether this region also interacts with Tat by co-IP assays with a panel of TRAF6 mutations (F471A, Y473A, and F471A/Y473A). None of these mutations affected the interaction (fig. S5B).

The structure of the Tat/P-TEFb complex and earlier biochemical studies showed that HIV-1 Tat forms a Zn-mediated bridge with Cys261 of CycT1 (*57*, *65*). Given the importance of the Tat cysteine residues for TRAF6-binding described above, we suspected that Tat may use a similar Zn-mediated bridge. We generated mutants of surface cysteines in the TRAF domain of TRAF6 (C349A, C366A, C390A, C403, and C497A). Among these mutants, C366A reduced the interaction with Tat, and C403A to a lesser extent (fig. S5C). We also observed slight decreases in NF-κB activation in mutants C366A and C403A of TRAF6 (fig. S5D). In comparison to wild-type TRAF6, there is no significant deficiency in K63-ubiquitin chain production or auto-ubiquitination in these two cysteine mutations (fig. S5, E and F), suggesting that the mutation of these two residues may not completely abolish the interaction between Tat and TRAF6. Given that single cysteine mutations could still retain Tat-mediated NF-κB activation, it implies the involvement of additional residues in the TRAF domain in the interaction. Apart from the Zn-mediated bridge, other residues such as Lys41 or Phe38 on Tat also contribute to the interaction with TRAF6, indicating a potential direct protein-protein interaction. This suggests a more intricate network of residues involved in the Tat-TRAF6 interaction, beyond the initially identified cysteine mutations. Further investigations into these additional residues and their roles in the interaction will provide a more comprehensive understanding of the molecular mechanisms.

### Tat-mediated NF-κB activation via TRAF6 is conserved among primate lentiviruses

**Fig. 6.**
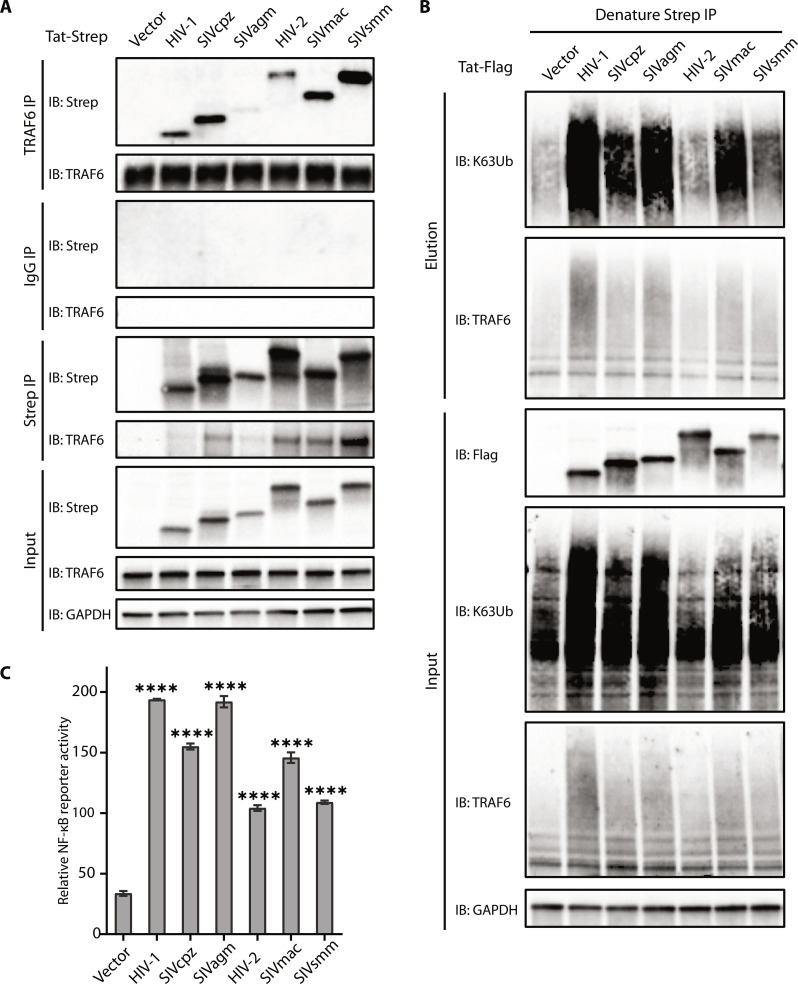
Stimulation of NF-κB activation by Tat through TRAF6 is conserved among primate lentiviruses. (**A**) Co-IP analysis [with anti-TRAF6 or immunoglobulin G (IgG)] and Western blot analysis (with anti-Strep) of HEK293T cells transfected with plasmids encoding Tat from HIV-1, SIVcpz, SIVagm, HIV-2, SIVmac, SIVsmm, or vector for 48 hours. (**B**) In vivo ubiquitination of wild-type or mutant TRAF6 expressed in HEK293T cells cotransfected with HA-ubiquitin and HIV-1, SIVcpz, SIVagm, HIV-2, SIVmac, SIVsmm Tat, or an empty vector. Cells were lysed under denaturing conditions, and IPs were performed with Strep-Tactin resin to detect ubiquitination by Western blot using an anti-ubiquitin antibody. (**C**) Luciferase reporter assays to measure NF-κB activation of HEK293T cells transfected with an empty vector or plasmids encoding Tat from HIV-1, SIVcpz, SIVagm, HIV-2, SIVmac, or SIVsmm together with TRAF6 for 24 hours. The graphs show the means ± SD of three biological replicates and were normalized to cotransfected NLuc activities.

Despite its small size (8 to 11 kDa), Tat contains numerous critical functional regions that are conserved among HIVs and SIVs. On the basis of sequence alignments, a variable sequence between 20 and 40 residues precedes the highly conserved cysteine-rich segment (fig. S6A), which contains key residues for NF-κB activation as described above. Co-IP experiments indicate that Tat proteins from other primate lentiviruses, including HIV-2 and SIVs (SIVcpz, SIVagm, SIVmac, and SIVsmm) also interact with both endogenous and ectopically expressed TRAF6 (Fig. 6A and fig. S6B). Since the TRAF domains of TRAF6 are highly conserved among primates (fig. S6C), the interaction between Tat and TRAF6 appears to occur similarly during various primate lentiviral infections. As with HIV-1 Tat, the in vivo ubiquitination assays indicate that overexpression of Tat proteins from other lentiviruses can also enhance auto-ubiquitination of TRAF6 and production of K63 ubiquitin chain to various degrees (Fig. 6B). The Tat proteins from different primate lentiviruses also stimulated NF-κB activation (Fig. 6C), consistent with their ability to enhance auto-ubiquitination and K63-ubiquitin chain production. Their activity levels differed among the SIVs: SIVagm exhibited relatively strong activation similar to that of HIV-1, whereas SIVsmm showed relatively weaker activation similar to HIV-2. To examine this difference, we found that HIV-1 Tat oligomerizes (or aggregates) much more strongly than HIV-2 based on sizing column data (fig. S6D), suggesting that the lower ubiquitination and TRAF6-related NF-kB activation of HIV-2 or SIVsmm Tat may be due to the inherent properties of the Tat proteins. SIVcpz and SIVmac both showed slightly weaker activation than HIV-1 but stronger than HIV-2 (Fig. 6C). This disparity may be due to their distinct evolution paths and differing abilities to stimulate NF-κB. These results suggest that Tat-mediated stimulation of NF-κB activation through TRAF6 has been functionally conserved during viral evolution, and presumably plays a crucial role in the infection of not just HIV-1, but also other lentiviruses.

## DISCUSSION

Primate lentiviruses are known to regulate the activation of NF-κB through different mechanisms to modulate viral gene expression and host immune responses. During the early phase of HIV-1 infection, activation of NF-κB is necessary to initiate basal transcription from the viral promoter, while at late stages, viral gene products down-regulate host innate responses, which would reduce viral replication or trigger adaptive immune responses. TRAF6 is a key regulator of canonical NF-κB signaling (*37*, *66*, *67*), which plays an important role in the immune response against pathogen infection (*2*). Therefore, TRAF6 can be a prime target for the virus to hijack. In the case of herpes simplex virus infection, the U_L_37 virion structural protein activates NF-κB by targeting TRAF6 (*68*). In tick-borne flaviviruses (TBFVs) infection, the viral nonstructural 3 (NS3) protein interacts with TRAF6 during infection to support TBFV replication (*69*).

**Fig. 7.**
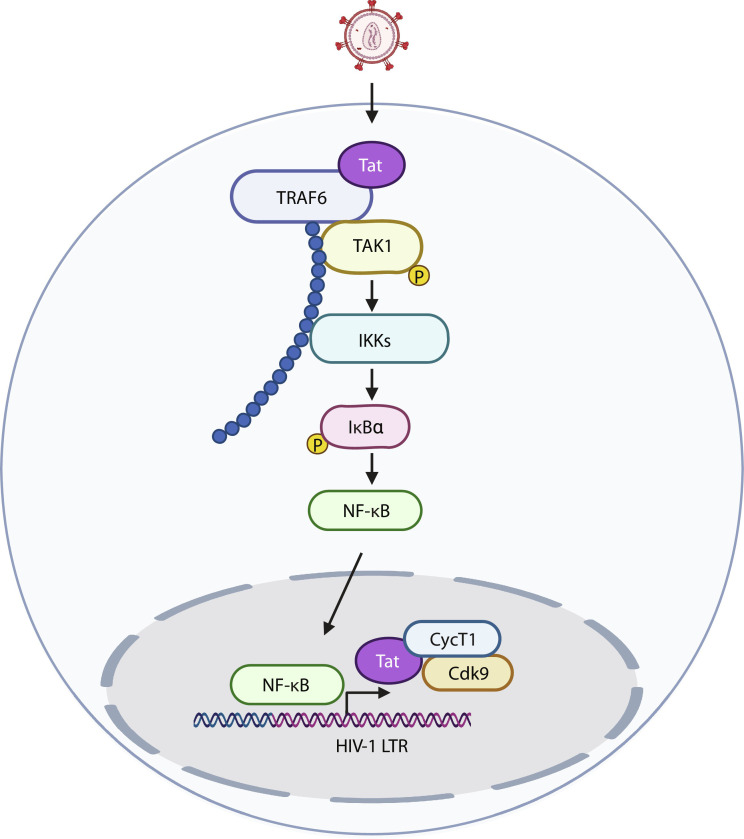
Model of Tat stimulation of TRAF6-mediated NF-κB activation. Tat directly interacts with TRAF6 to promote its ubiquitination and activate NF-κB to further stimulate viral transcription.

HIV-1 transcription is stimulated by the activation of the canonical NF-κB pathway via its two NF-κB sites in the viral LTR to increase basal transcription. Subsequently, Tat recruits P-TEFb to the TAR region of the LTR to phosphorylate RNA Pol II and enhance integrated viral genome transcription elongation from the integrated proviral genome (*15*, *17*). Besides P-TEFb, Tat also recruits a diverse series of transcriptional complexes, including enzymes with histone acetyltransferases (HATs) activity to induce chromatin remodeling of proviral genes, which also contributes to the increase in proviral transcription rate (*70*, *71*). Tat activation also involves the direct activation of NF-κB via various mechanisms mostly in the downstream part of the NF-κB pathway: through the double-stranded PKR and PKC (*24*, *27*, *28*); via physical interaction with key regulator proteins such as IκB-α and p65 (*25*); and by enhancing p50 acetylation by CBP/p300 complex to increase transcriptional activity of the NF-κB complex (*26*). In this study, we establish a novel mechanism of Tat-mediated NF-κB activation via a direct interaction between Tat and TRAF6, leading to the enhancement of TRAF6 ubiquitination and further stimulation of viral transcription (Fig. 7). Because the knockdown of TRAF6 hampers both HIV-1 replication and latency reactivation, we propose that the involvement of Tat-mediated NF-κB activation is significant in both processes. Combined with previous studies on the activation of NF-κB by Tat, we speculate that cytosolic Tat can activate NF-κB at different levels (both upstream and downstream of TAK1) to increase NF-κB–dependent transcription, while nuclear Tat enhances viral transcription elongation through TAR and the P-TEFb complex.

As a small viral protein, Tat appears to be largely unfolded without host factor binding, potentially allowing multiple partners to organize different Tat complexes. The cysteine-rich region and its binding to zinc are instrumental in its folding, potentially making it a hot spot for interactions with different host factors (*57*, *65*, *72*–*74*). Here, we found that this region is also important for NF-κB activation. Since this region is highly conserved among SIVs and HIVs, it is reasonable to speculate that the cysteine-rich region has co-evolved with factors responsible for both HIV-1 transcription and NF-κB activation to optimize the viral life cycle.

Notably, the interaction between the cysteine-rich region of Tat and TRAF6 has the potential to trigger TRAF6 oligomerization, a process that enhances auto-ubiquitination and leads to the production of K63-linked ubiquitin chains, both contributing to NF-κB activation. However, our data indicate that the interaction between Tat and TRAF6 is necessary but not sufficient for NF-κB activation. For example, a F38A Tat mutant maintained the interaction with TRAF6 but showed much weaker NF-κB activation. Another notable contrast is observed between HIV-1 and HIV-2 Tat, where HIV-2 Tat displayed diminished ubiquitination activity and NF-κB activation, correlating with its weaker oligomerization or aggregation behavior and hinting at varying capabilities of NF-κB activation between these two viruses.

In addition to TRAF6, there are other Tat-interacting host factors related to the NF-κB pathway, such as UBE2O and ZFP91 (*29*, *30*). UBE2O, which is involved in the regulation of Tat transcriptional activity (*30*), can inhibit NF-κB activation by preventing the polyubiquitination of TRAF6 (*31*). Our results indicate that Tat enhances TRAF6-dependent NF-κB activation in a UBE2O-independent manner. Since UBE2O has inhibitory effects on TRAF6, it is tempting to speculate that Tat may use UBE2O to down-regulate NF-κB activation to suppress viral transcription during HIV-1 latency. ZFP91, a noncanonical NF-κB signaling pathway regulator, is also important for Tat-dependent transcription (*30*, *75*). However, it is not known if Tat affects the noncanonical NF-κB signaling pathway through ZFP91 during HIV-1 latency. Further studies will unravel the cross-talk between activation and inactivation of NF-κB through different Tat host factors and possible roles in HIV-1 replication or latency.

## MATERIALS AND METHODS

### Cell culture, plasmids, and reagents

HeLa [American Type Culture Collection (ATCC), catalog no. CCL-2], HEK293T cells (ATCC, catalog no. CRL-3216), and NIH/3 T3 (ATCC, catalog no. CRL-1658) were maintained in Dulbecco’s modified Eagle’s medium [10% fetal bovine serum (FBS) and 1% Pen-Strep] at 37°C with 5% CO_2_. Jurkat E6-1 (ATCC, catalog no. TIB-152) and J-Lat6.3 (HIV Reagent Program, RRID: CVCL_8280) cells were maintained in RPMI 1640 (10% FBS and 1% Pen-Strep) at 37°C with 5% CO_2_.

The cDNAs for all proteins used in this study were cloned into pcDNA4/TO (Thermo Fisher Scientific) unless otherwise noted. *TRAF6*, *TRAF2*, *TRAF3*, *MyD88*, *TAK1*, *IKK*β, *IκB*α, and *p65* genes were obtained from Addgene (#66929, #44104, #44032, #12287, #23693, #111195, #21985, and #21966), and subcloned into pcDNA4/TO (Thermo Fisher Scientific) with a C-terminal 3xFlag tag or 2xStrep tag. UBE2O, Cdk9, CycT1, HIV-1 HXB2 Tat, and human ubiquitin were constructed as previously described (*55*). All TRAF6 and HIV-1 Tat mutants were generated by standard quick-change mutagenesis. HIV-2 *Tat* and SIV *Tat* (agm.ver, cpz, mac, and smm) were synthesized as a gBlock gene fragment (IDT) and subcloned into pcDNA4/TO (Thermo Fisher Scientific) with a C-terminal 3xFlag tag or 2xStrep tag. NF-κB reporter plasmid (pGL4.32 [*luc2P*/NF-κB-RE/Hygro] Vector, E8491) and the control plasmid (pNL1.1.TK [*Nluc*/TK] Vector, N1501) were purchased from Promega.

*UBE2N* and *Uev1a* were synthesized as a gBlock gene fragment (IDT) with appropriate restriction sites and cloned into pGEX6p-1 (Cytiva). The cDNA of full-length *TRAF6* was inserted into pFastBac (Addgene, #48294) with a 2xStrep tag fused at the C termini of the proteins. The coding sequences of TRAF6 (CC + TRAFC residues 333 to 528, TRAFC residues 346 to 504) with an N-terminal PreScission Protease Cleavage site and MBP-tag generated by two-step PCR were cloned into pET21b (+) (Novagen). The coding sequences of HIV-1 HXB2 Tat (residues 1 to 20, residues 1 to 40, and residues 1 to 72) with a C-terminal 2xStrep tag were cloned into pGEX6p-1 (Cytiva).

For HeLa cell and HEK293T cell expression, DNA was transfected with PolyJet (SignaGen), and siRNA was transfected with HiPerFect (Qiagen) or RNAiMAX (Thermo Fisher Scientific). For Jurkat E6-1 cells, DNA or siRNA was transfected by the Neon Transfection System (Thermo Fisher Scientific). All transfections were performed according to the manufacturer’s protocol. The Nano-Glo Dual-Luciferase Reporter Assay System was purchased from Promega (N1610). Laemmli buffers (2× and 4×) were purchased from Bio-Rad. NE-PER Nuclear and Cytoplasmic Extraction Reagents (Thermo Fisher Scientific, 78833) were used to separate nuclear and cytoplasmic fractions. All antibodies are listed in table S1.

### Dual-luciferase reporter assay

HEK293T cells were seeded into 96-well plates at a density of 3 × 10^4^ per well and cultured for 24 hours. Then, 200 ng of plasmid containing 5 ng of Tat, 1.25 ng of wild-type or mutant TRAF6, 49.5 ng of NF-κB reporter plasmid (pGL4.32), and 0.5 ng of pNL1.1.TK [Nluc/TK] control plasmid were cotransfected with PolyJet (SignaGen) following the manufacturer’s instructions. Each sample was performed in biological triplicates or quadruplicates. After 24 hours of transfection, the cells were harvested. The dual-luciferase reporter assay (Promega) and a microplate reader (Ultra Evolution, TECAN) were used to measure Fluc and NLuc activities according to the manufacturer’s protocol (Promega). The reporter gene activity was determined on the basis of the normalization of FLuc activity to NLuc activity.

### HIV reporter assay

One nanogram of wild-type or mutant Tat-expressing plasmid and 5 ng of wild-type or mutant TRAF6-expressing plasmid were cotransfected with 20 ng of HIV-1 LTR-driven firefly luciferase reporter plasmid (*17*) and 0.2 ng of control plasmid pNL1.1.TK into HEK293T cells. After 48 hours, cells were lysed in 1× passive lysis buffer (Promega), and FLuc and NLuc luciferase activities were measured with the Promega Nano-Glo Dual-Luciferase Reporter Assay System and microplate reader (Ultra Evolution, TECAN). Tat-dependent FLuc values were normalized to NLuc values. The assay was performed in biological triplicates or quadruplicates.

### RNAi knockdown of Tat’s host factors

HEK293T cells were transfected with N.S. or specific siRNAs at 5 to 10 nM final concentrations using HiPerFect. After 48 hours, cells were transfected with 1 ng of Tat-expressing plasmid, 50 ng of pNL4-3 ∆ENV ∆Tat ∆Nef + GFP (*55*), and 10 ng of CMV-firefly luciferase plasmid as a transfection control using PolyJet (SignaGen). After an additional 48 hours, cells were lysed in cell lysis buffer [5 mM tris-HCl (pH 8.0), 85 mM KCl, and 0.5% IGEPAL CA-630]. Cell-associated HIV-1 p24 production was detected by p24 ELISA. In parallel, 10 μl of lysate was used for standard luciferase assay. All p24 values were normalized by firefly luciferase activity for each siRNA to generate a relative Tat activity score, defined as [p24^host RNAi^/FFL^host RNAi^]/[p24^N.S. RNAi^/FFL^N.S. RNAi^]. For analysis of protein knockdown, 2× Laemmli sample buffer was added to siRNA-treated cells and then loaded onto gels. Knockdowns were determined by Western blot with the indicated antibodies.

For Jurkat T cells, siRNA, and plasmids were transfected with Neon Transfection System (Thermo Fisher Scientific). 2 × 10^5^ Jurkat T cells with 1 μl of siRNA (100 μM) in a total volume of 10 μl of Resuspension Buffer R were electroporated with three pulses of 1325 V for 10 ms each. The transfected cells were cultured in 24-well plates containing 500 μl of prewarmed RPMI 1640 medium with 10% FBS and no antibiotics. After 48 hours, cells were transfected with 4 μg of DNA (1 μg of Tat-expressing plasmid, 2 μg of pNL4-3 ∆ENV ∆Tat ∆Nef + GFP, and 1 μg of CMV-firefly luciferase plasmid) by the Neon Transfection System with the same voltage, pulse width, and number of pulses as siRNA transfection. After an additional 48 hours, cells underwent the same procedure as for HEK293T cells above. siRNAs are the following:

UBE2O #1 (Qiagen, SI04153863)

UBE2O #2 (J-008979–08)

TRAF6: GCTTGATGGCATTACGAGAAGCAGT (IDT, hs.Ri. TRAF6.13.2)

TRAF6: ON-TARGETplus SMARTpool (Dharmacon, M-004712-00)

Cdk9: TAGGGACATGAAGGCTGCTAA (Qiagen, SI00605066)

CycT1: AGGCTTTGAACTAACAATTGA (Qiagen, SI02625707)

Non-silencing (N.S.): Qiagen All-Star negative control (SI03650318)

### Denaturing in vivo ubiquitination assay

HEK293T cells were transfected with 5 μg of total DNA containing a plasmid expressing Strep-tagged wild-type or mutant TRAF6, Flag-tagged Tat, and hemagglutinin (HA)–ubiquitin using PolyJet. After 48 hours, cells were lysed in an equal volume SDS lysis buffer [2% SDS, 50 mM tris-HCl (pH 8.0), 150 mM NaCl, and 0.5 mM dithiothreitol (DTT)] and boiled for 10 min. Boiled lysates were sonicated by using Fisher Sonic Dismembrator Model 500 and centrifuged at 15,000 rpm for 10 min. Cleared lysates were incubated with Strep-Tactin Superflow resin (IBA LifeScience) for 3 hours at room temperature. The resin was washed with radioimmunoprecipitation assay buffer and the bound material was eluted with 1× Strep-Tactin elution buffer with desthiobiotin (IBA LifeScience). Ubiquitination of TRAF6 was detected by immunoblotting with anti-HA- or anti-Lys63-specific ubiquitin antibodies.

### Expression and purification of recombinant proteins

Recombinant proteins (UBE2N and Uev1a, USP5 (163-291a.a), ubiquitin with an N-terminal HA tag, and C-terminally truncated HIV-1 Tat with 2xStrep tag cloned in the pGEX6p-1 vector) were expressed as fusion proteins with an N-terminal GST tag in *Escherichia coli* BL21 (DE3). The GST-fusion proteins were digested on columns using PreScission protease. The released ubiquitin proteins were collected and further purified by Superdex 200 Increase (Cytiva). TRAF6 full-length protein with a 2xStrep tag was expressed in insect SF9 (*Spodoptera frugiperda*) cells and purified by Strep-tag affinity chromatography and size exclusion chromatography. C-terminally truncated TRAF6 with N-terminal MBP tag and C-terminal 6xHis tag was expressed in *E. coli* BL21 (DE3) and purified by using nickel affinity chromatography followed by MBP affinity chromatography.

### In vitro ubiquitination assay

Assays were performed in a total volume of 30 μl with 0.2 μM recombinant human E1, 0.6 μM E2(UBE2N/ Uev1a), 0.2 μM TRAF6, 5 μM HA-tagged ubiquitin, and increasing amounts of Tat proteins (0.025, 0.05, 0.1, and 0.2 μM) in the reaction buffer containing 50 mM tris-HCl (pH 7.4), 100 mM NaCl, 1 mM DTT, 5 mM MgCl_2_, and 4 mM adenosine 5′-triphosphate at 37°C for 1 hour. Reactions were stopped with 4× Laemmli sample buffer (containing 2-mercaptoethanol) before SDS–polyacrylamide gel electrophoresis (PAGE). The K63-linked ubiquitination chain was verified by immunoblotting with an anti-ubiquitin (K63 linkage–specific) antibody.

### Gel filtration

HEK293T cells were lysed in lysis buffer [50 mM tris-HCl (pH 8.0), 150 mM NaCl, 1.5 mM MgCl_2_, 0.2 mM EDTA, 1% IGEPAL CA-630, and protease inhibitor cocktail]. Cell extracts were filtered through a 0.22-μm filter and loaded on a Superose 6 Increase 10/300 GL (Cytiva) column in 1× phosphate-buffered saline containing 1 mM DTT. The flow rate was 0.5 ml/min and 0.5 ml of fractions was collected after elution of the void volume. Each fraction was subjected to Western blot analysis.

### Subcellular fractionation

Subcellular fractionation was performed using the NE-PER buffer kit (Thermo Fisher Scientific) per the manufacturer’s instructions. A Bradford assay (Bio-Rad) was used to determine the protein concentration in each fraction. Ten micrograms of protein from each fraction was analyzed by SDS-PAGE and validated using nuclear and cytoplasmic markers SP1 or α-tubulin, respectively.

### Affinity purification and Western blot

HEK293T cells were transfected with 5 μg of total plasmid DNA encoding Strep-tagged or Flag-tagged bait protein. After 48 hours, cells were collected, lysed in lysis buffer [Strep lysis buffer: 50 mM tris-HCl (pH 7.5), 150 mM NaCl, 2 mM MgCl_2_, 0.5% Triton X-100, 0.2% Na-deoxycholate, and protease inhibitor cocktail (Roche); Flag lysis buffer: 50 mM tris-HCl (pH 8.0), 150 mM NaCl, 1.5 mM MgCl_2_, 0.2 mM EDTA, 1% IGEPAL CA-630, and protease inhibitor cocktail] and incubated for 30 min at 4°C by gentle rocking. Lysate was cleared and incubated with Strep-Tactin Superflow resin (IBA LifeScience) or magnetic M2-Flag resin slurry (Sigma-Aldrich) at 4°C. The resin was washed with WASH buffer [Strep WASH buffer: 50 mM tris-HCl (pH 7.5), 150 mM NaCl, 2 mM MgCl_2_, and 0.5% Triton X-100; Flag WASH buffer: 50 mM tris-HCl (pH 8.0), 150 mM NaCl, 1.5 mM MgCl_2_, 0.2 mM EDTA, and 0.1% IGEPAL CA-630] and eluted with 1× Strep elution buffer (IBA-GmbH) diluted in Strep WASH buffer or Flag elution buffer (200 μg/ml 3xFlag peptide, diluted in Flag WASH buffer) by gentle shaking at room temperature for 30 min. The eluate was mixed 1:1 with the 2× Laemmli sample buffer, boiled, and loaded on an SDS-PAGE gel. Proteins were detected with indicated antibodies. The Materials and Methods section should provide sufficient information to allow replication of the results: begin with the “Experimental design” section describing the objectives and design of the study as well as prespecified components.

### Endogenous IP

HEK293T cells were lysed in lysis buffer [50 mM tris-HCl (pH 8.0), 150 mM NaCl, 2 mM MgCl_2_, 0.5% Triton X-100, 0.2% Na-deoxycholate, and Roche protease inhibitor cocktail] for 30 min at 4°C by gentle rocking. Cleared cell lysates were incubated with control immunoglobulin G or TRAF6 antibodies (1 to 10 μg) for 3 hours. Protein A Dynabeads slurry (Invitrogen, 10001D) was washed and added to the antibody-lysate mixture for an additional 3 hours. Dynabeads were then washed with WASH buffer [50 mM tris-HCl (pH 8.0), 150 mM NaCl, 2 mM MgCl_2_, and 0.5% Triton X-100]. For elution, 45 μl of 2× Laemmli buffer (Bio-Rad) was added to the beads and incubated at 65°C for 15 min, with occasional mixing. After elution, 1.5 μl of 2-mMercaptoethanol was added to the eluate, which was then processed by SDS-PAGE and Western blotting.

### Flow cytometry

J-Lat6.3 cells with or without TRAF6 siRNA treatment were cultured in a 24-well plate at 1 × 10^5^ cells per well in a total volume of 200 μl of RPMI 1640 media supplied with 10% FBS. Cells were treated with TNF-α (10 ng/ml), PMA (0.25 μM), or dimethyl sulfoxide for 18 hours. GFP-positive cells were gated and analyzed by flow cytometry using an Attune NxT Flow Cytometer (Thermo Fisher Scientific).

### HIV-1 infection of Jurkat T cells

NL4-3 (Tat-SF) virus was generated as described (*76*). The infection of Jurkat T cells was performed by spinoculation for 1 hour at 1200 rcf with NL4-3 (Tat-SF) virus at MOIs of 5 to 10 in the presence of PEI (1 μg/ml; Polysciences) and polybrene (8 μg/ml; Millipore). For TRAF6 knockdown experiments, Jurkat cells that had been transfected with siRNA for 48 hours were then infected with the virus for an additional 56 hours before harvesting. Endogenous TRAF6 IP was performed as described above.
