# Supplementary material for: Enhanced NF-κB activation via HIV-1 Tat-TRAF6 cross-talk
Source: Sci Adv. 2024 Jan 19;10(3):eadi4162. doi: 10.1126/sciadv.adi4162 (PMC10798561; doi:10.1126/sciadv.adi4162)
Supplement: Supplementary file 1 — Figs. S1 to S6 Table S1 Legends for data files S1 to S3 [file sciadv.adi4162_sm.pdf]

Science Advances
AAAS

Supplementary Materials for

Enhanced NF-κB activation via HIV-1 Tat-TRAF6 cross-talk

Yang Li *et al.*

Corresponding author: Alan D. Frankel, frankel@cgl.ucsf.edu

Sci. Adv. **10**, eadi4162 (2024)
DOI: 10.1126/sciadv.adi4162

The PDF file includes:

Figs. S1 to S6
Table S1
Legends for data files S1 to S3

Other Supplementary Material for this manuscript includes the following:

Data files S1 to S3

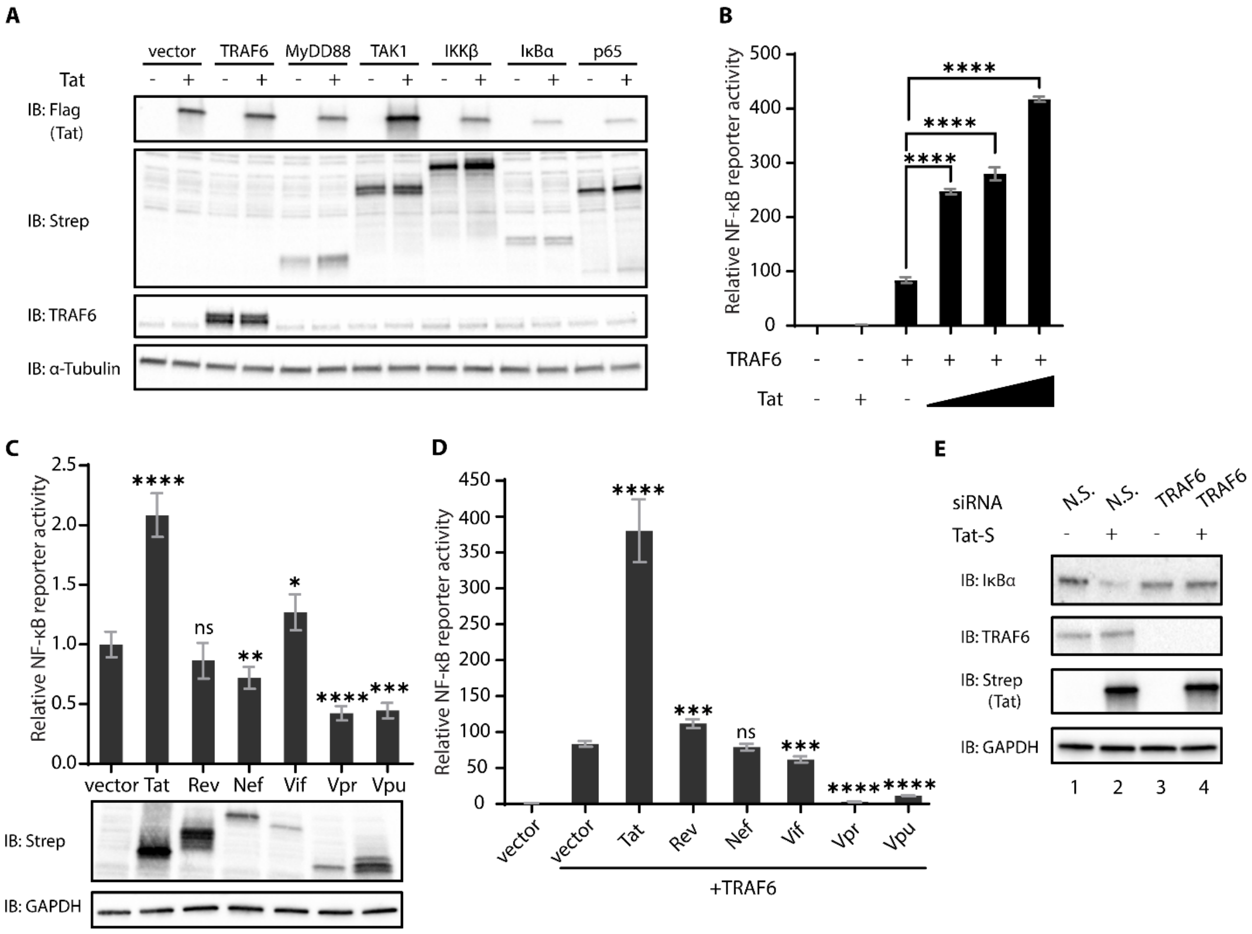

Fig. S1. Tat enhances NF-κB activation through TRAF6. (A), Western blot analysis of the protein levels from Fig. 1B using Flag, Strep, TRAF6 or α-Tubulin antibodies. **(B)**, HEK293T cells were transfected with an NF-κB luciferase reporter and the internal control NLuc, as well as a control empty vector, TRAF6 or increasing amounts (wedge) of Tat-encoding plasmid for 24 hr. NF-κB activation was measured by Nano-Glo Dual-luciferase reporter assay. **(C)**, (top) Luciferase reporter assays to measure NF-κB activity in HEK293T cells transfected with an empty vector or plasmids encoding HIV-1 Tat, Rev, Nef, Vif, Vpr, or Vpu for 24 hr, and treated with TNFα (5 ng/mL) for 12 hr. (bottom) Anti-Strep western blot analysis of whole cell lysate of viral proteins. **(D)**, Luciferase reporter assays to measure NF-κB activation in HEK293T cells transfected with an empty vector or plasmids encoding HIV-1 Tat, Rev, Nef, Vif, Vpr or Vpu together with an empty vector or the plasmid encoding TRAF6 for 24 hr. **(E)**, Western blot analysis of IκBα in cells transfected with non-silencing (N.S.) and TRAF6 RNAi, 48 hr later followed by transfection with an empty vector or HIV-1 Tat expression plasmid for 24 hr. The graphs in (B) show the mean −/+ SD of three biological replicates and were normalized to co-transfected NLuc activities. The graphs in (D) and (E) show the mean −/+ SD of four biological replicates and were normalized to co-transfected NLuc activities. ns $p > 0.05$, $*p < 0.05$,

**p < 0.01, ***p < 0.001, ****p < 0.0001, and statistical significance was assessed by a two-tailed unpaired Student's t test.

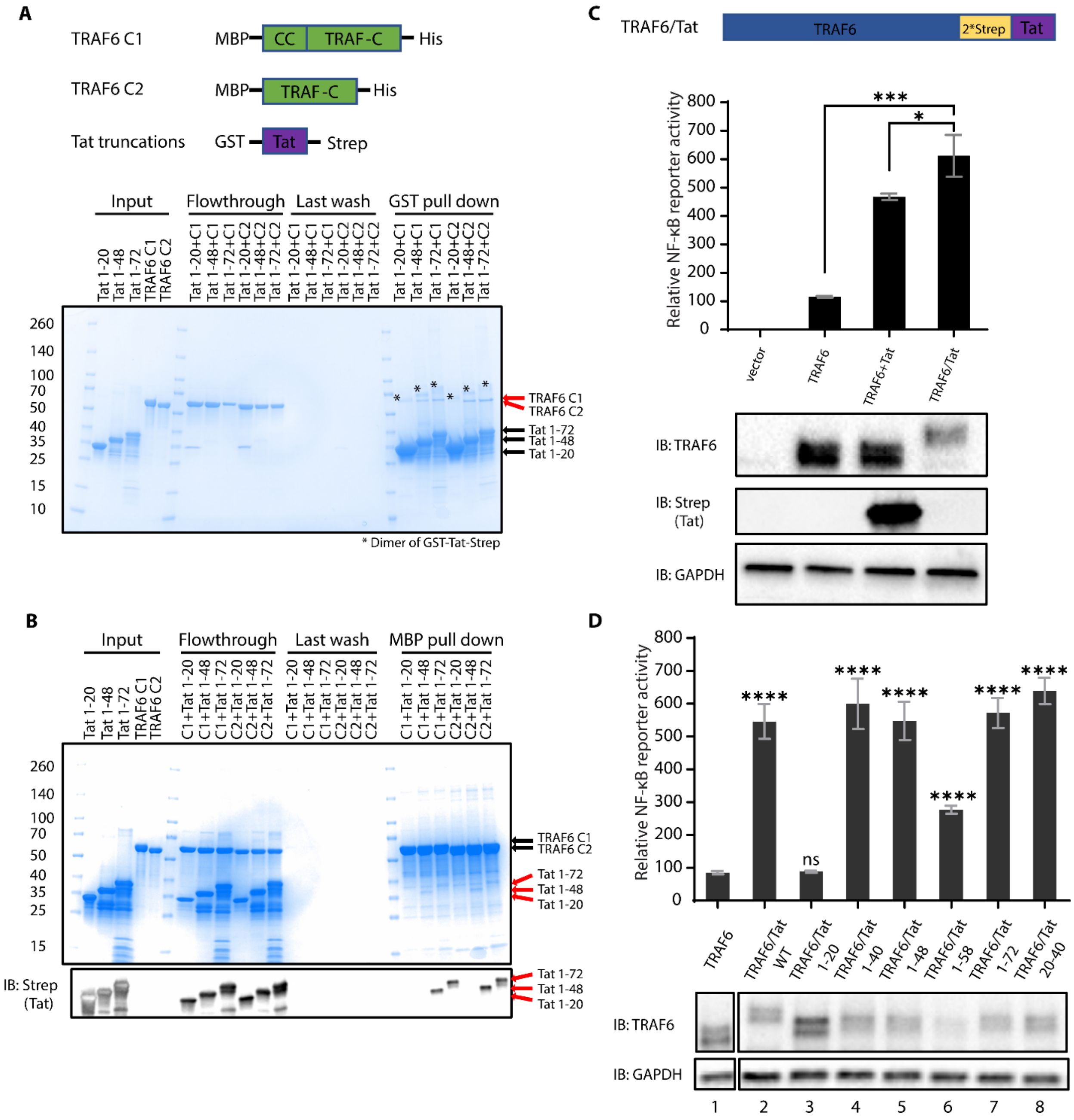

Fig. S2. The cysteine rich region of Tat is the minimal region for interacting with TRAF6 and enhancing NF-κB activation. (A), (top) Schematic representation of constructs for GST fusion proteins and domain representation of TRAF6 truncations used in the pull-down experiments. (bottom) *In vitro* GST pull-down experiments of GST-Tat truncations and MBP-TRAF6 C-6xHis. **(B)**, *In vitro* MBP pull-down experiments of MBP-TRAF6 C-6xHis and GST-Tat truncations. **(C)**, (top) Schematic diagram of the single-chain construct TRAF6-2xStrep-Tat. (bottom) Luciferase reporter assays to measure NF-κB activity in HEK293T cells transfected with an empty vector or plasmids encoding TRAF6, TRAF6 with Tat, or TRAF6-2xStrep-Tat for 24 hr. **(D)**, Luciferase reporter assays to measure NF-κB activity of HEK293T cells transfected

with plasmids encoding TRAF6 or TRAF6-2xStrep-Tat for 24 hr. The graphs in (C) show the mean −/+ SD of three biological replicates and were normalized to co-transfected NLuc activities. The graphs in (D) show the mean −/+ SD of four biological replicates and were normalized to co-transfected NLuc activities. ns $p > 0.05$, *$p < 0.05$, **$p < 0.01$, ***$p < 0.001$, ****$p < 0.0001$, and statistical significance was assessed by a two-tailed unpaired Student's t test.

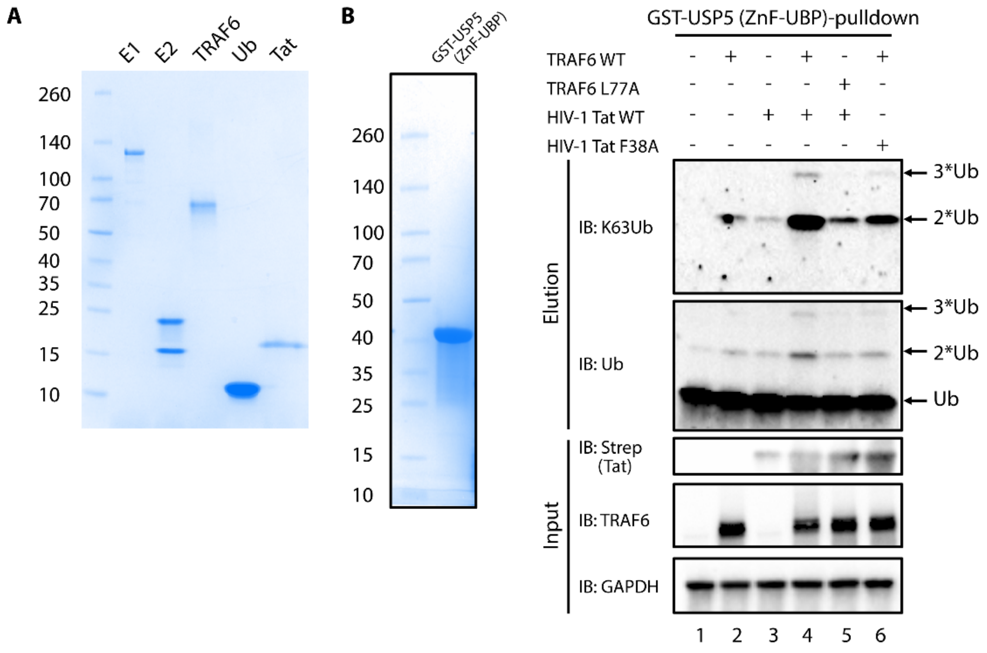

Fig. S3. Tat enhances the synthesis of K63-linked polyubiquitin chains *in vivo*. (A), Coomassie-stained gel of the purified proteins (E1, E2(UBE2N/ Uev1a), TRAF6, ubiquitin and Tat) that were used in the *in vitro* ubiquitination assay in Fig. 3A. **(B)**, (left) Coomassie-stained gel of the purified GST-USP5 (ZnF-UBP) that was used in the *in vivo* pulldown, (right) *In vivo* GST-USP5 (ZnF-UBP) pulldown of whole cell lysates of HEK293T cells transfected with either wild-type TRAF6 or TRAF6 L77A with or without Tat or Tat F38A.

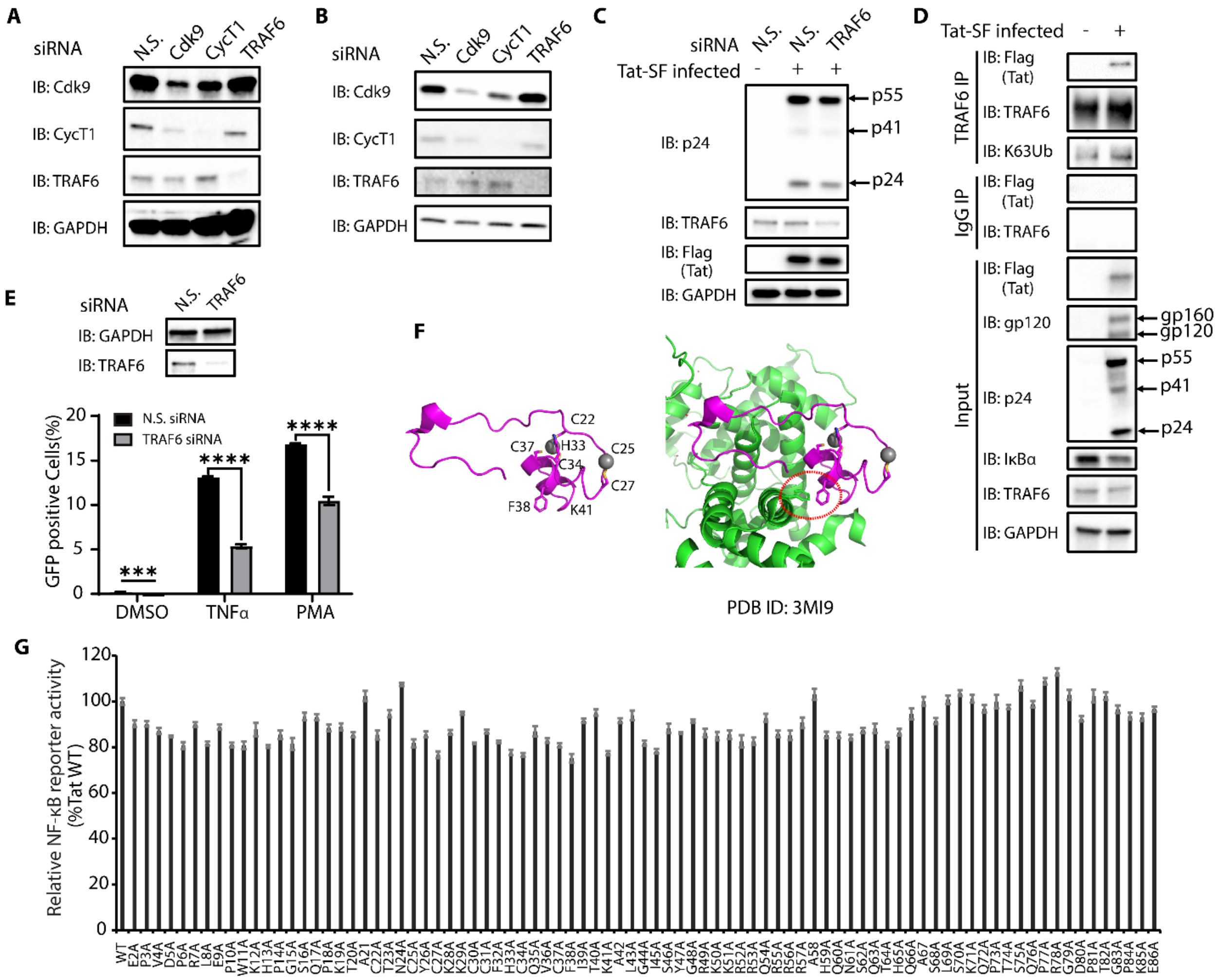

Fig. S4. The region required for the interaction between TRAF6 and Tat is highly conserved. (A), Western blots demonstrate specific protein knockdown in HEK293T cells for the indicated siRNAs used in Fig. 4C. **(B)**, Western blots demonstrate specific protein knockdown in Jurkat cells for the indicated siRNAs used in Fig. 4C. (**C**), Western blot analysis of p24 in Jurkat cells transfected with non-silencing (N.S.) and TRAF6 RNAi, 48 hr later followed by infection with NL4-3(Tat-SF) virus for 56 hr. (**D**), Co-IP analysis (with anti-TRAF6 or IgG) and western blot analysis (with anti-Flag, K63Ub, p24, gp120 and IκBα) of Jurkat cells infected with NL4-3(Tat-SF) virus for 56 hr. (**E**), (top) Western blot analysis of TRAF6 protein expression in J-Lat6.3 cells, (bottom) Reactivation of the latent HIV-1 reporter as measured by GFP flow cytometry. The knockdown of TRAF6 suppresses HIV-1 reactivation under basal conditions and upon activation with TNFα or PMA. The average of three independent experiments analyzed in triplicate −/+ SD is shown. ***$p < 0.001$, ****$p < 0.0001$, and statistical significance was assessed by a two-tailed unpaired Student's t test, **(F)**, All residues of HIV-Tat involved in TRAF6-binding are shown. **(G)**, Luciferase reporter assays to measure NF-κB activities in HEK293T cells transfected with wild-type or mutant Tat for 24 hr. The graphs show

the mean −/+ SD of four biological replicates and were normalized to co-transfected NLuc luciferase activities. The statistical significance of (G) is in the Supplementary file 1.

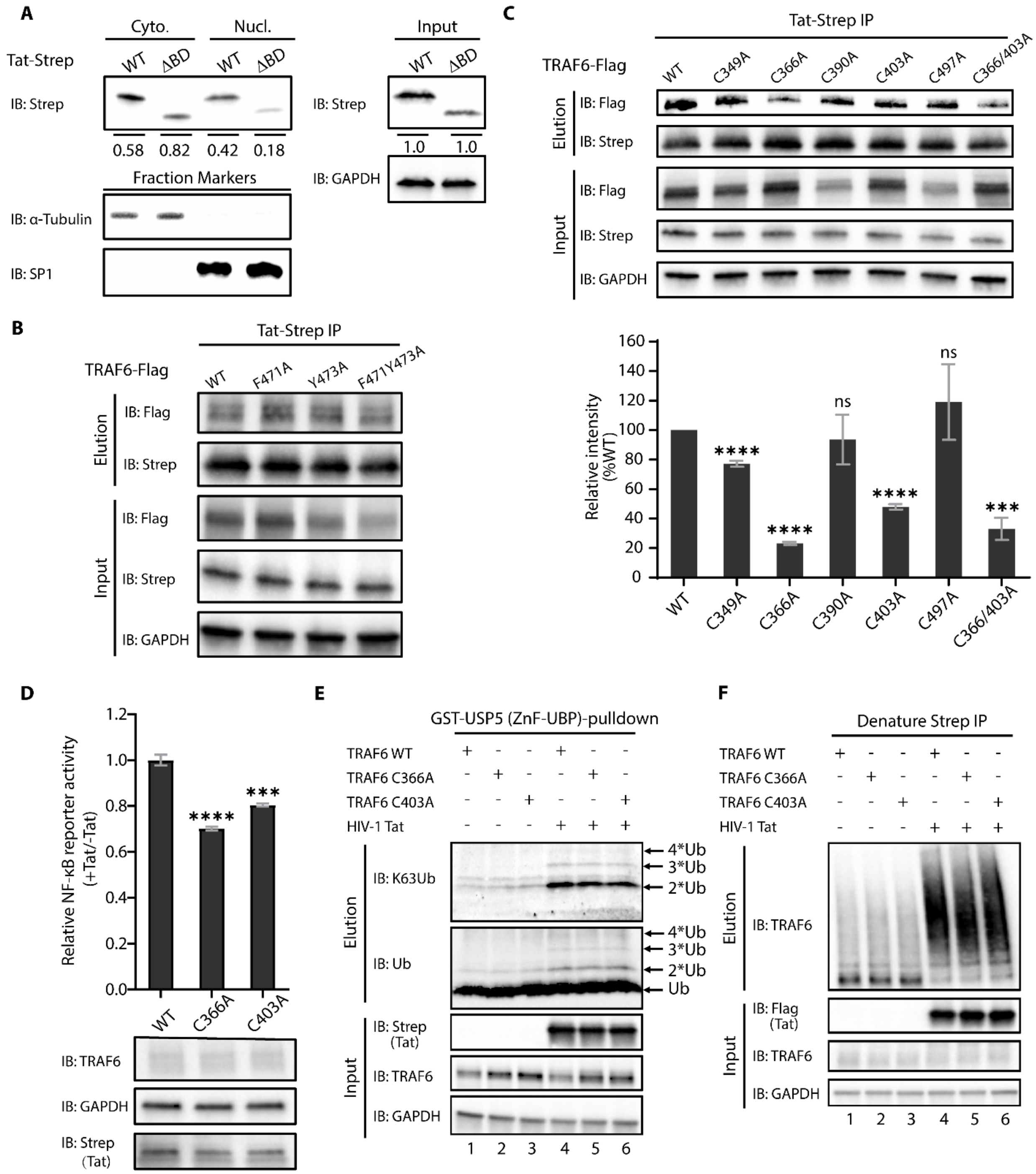

Fig. S5. Tat interacts with TRAF6 mainly through surface cysteines in the TRAF domain. **(A)**, Subcellular localization of wild-type or ΔBD Tat-S expressed in HEK293T cells was determined by fractionating the whole cell lysates into cytoplasmic (Cyto.) and nuclear (Nucl.) fractions. Numbers represent intensities of the bands corresponding to wild-type or ΔBD Tat relative to input levels of the same fraction, respectively. Tubulin and SP1 were used as markers for each fraction and show the quality of the separations. **(B)**, Co-IP assay to examine the

association of wild-type or mutant (F471A, Y473A, F471/Y473A) TRAF6 and HIV-1 Tat. **(C)**, Co-IP assay to examine the association of wild-type or cysteine mutants (C349A, C366A, C390A, C403A, C497A, C366/403A) TRAF6 and HIV-1 Tat. Quantification of TRAF6 levels is shown in the bottom panel, normalized to wild-type TRAF6. **(D)**, Luciferase reporter assays to measure NF-κB activity of HEK293T cells transfected with plasmids encoding wild-type or cysteine mutants (C366A, C403A) TRAF6 and HIV-1 Tat for 24 hr. The graphs show the mean −/+ SD of four biological replicates. ***$p < 0.001$, ****$p < 0.0001$, and statistical significance was assessed by a two-tailed unpaired Student's t test. **(E)**, *In vivo* GST-USP5 (ZnF-UBP) pulldown of whole cell lysates of HEK293T cells transfected with either wild-type or cysteine mutants (C366A, C403A) TRAF6 with or without Tat. Ubiquitin chains were detected by western blot with anti-ubiquitin (K63-linkage specific) antibody. **(F)**, *In vivo* ubiquitination of wide-type or mutant TRAF6 in HEK293T co-transfected with HA-ubiquitin together with an empty vector or Tat-encoding plasmid. Cells were lysed under denaturing conditions, and IPs were performed with Strep-Tactin resin to detect auto-ubiquitination by western blot with anti-TRAF6 antibody.

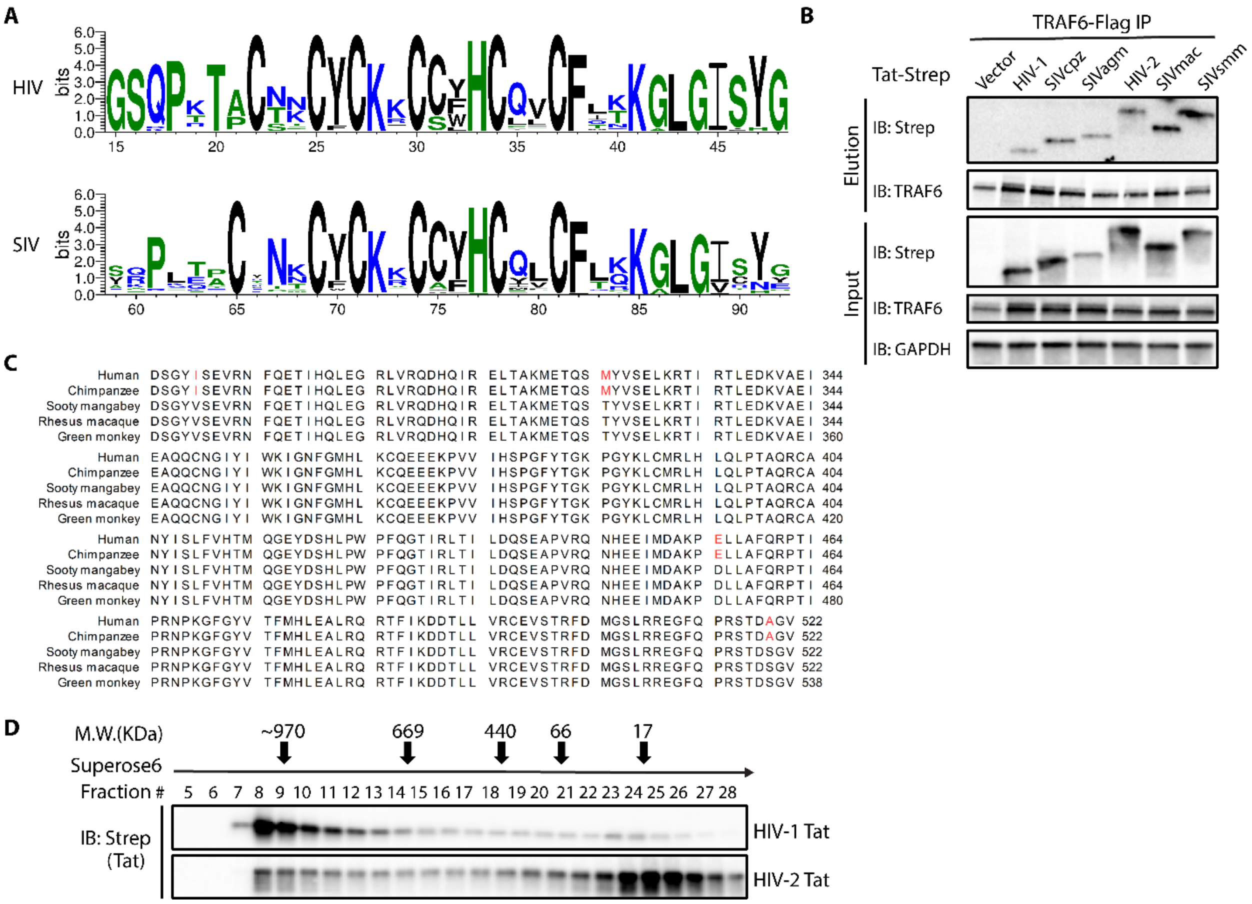

Fig. S6. Tat's cysteine-rich region and TRAF6's TRAF domain are highly conserved among primate lentiviruses and their hosts. (A), Sequence logos showing conservation of the corresponding cysteine-rich regions of Tat from HIV and SIV were computed from their sequence alignments (extracted from 2018 curated amino acid alignments of HIV-1/SIVcpz, HIV-2/SIVsmm, and other SIV from the Los Alamos HIV database (www.hiv.lanl.gov)). **(B)**, Co-IP analysis (with anti-Flag) and western blot analysis (with anti-Flag or anti-Strep) of HEK293T cells transfected with plasmids encoding Flag-tagged TRAF6 and plasmids encoding Tat from HIV-1, SIVcpz, SIVagm, HIV-2, SIVmac, SIVsmm or vector for 48 hr. (**C**), Sequence alignment of the TRAF domain among primate TRAF6 proteins. (**D**), Whole cell lysates of HEK293T cells transfected with plasmids encoding Strep-tagged HIV-1 Tat or HIV-2 Tat for 48 hr were prepared and separated on a Superose 6 Increase 10/300 GL gel filtration column. Fractions were collected and subjected to SDS-PAGE and probed with an anti-Strep antibody. Fraction numbers are indicated at the top of the gel. Molecular mass markers were run under the same conditions and are shown on top with arrows corresponding to the fraction that represents the peak of their elution profile.

Table S1. Antibodies used for western blots or IPs

Antibody	Source	Identifier
TRAF6	Cell Signaling Technology	Cat# 8028, RRID: AB_10858223
TRAF6	Biolegend	Cat# 654502, RRID: AB_2561868
IκBα	Cell Signaling Technology	Cat# 4814, RRID: AB_390781
CycT1	Santa Cruz Biotechnology	Cat# sc-271348, RRID: AB_10608086
Cdk9	Santa Cruz Biotechnology	Cat# sc-13130, RRID: AB_627245
Sp1	Santa Cruz Biotechnology	Cat# sc-14027, RRID: AB_2171049
Ubiquitin	Santa Cruz Biotechnology	Cat# sc-8017, RRID: AB_628423
GAPDH	Proteintech	Cat# 60004-1-Ig, RRID: AB_2107436
Flag	Sigma	Cat# F1804, RRID: AB_262044
α-Tubulin	Sigma	Cat# T5168, RRID: AB_477579
Strep-HRP	Millipore	Cat# IMG-71591, RRID: AB_613665
Goat anti-rabbit IgG, HRP	Abcam	Cat# ab205718, RRID: AB_2819160
Goat anti-Mouse IgG, HRP	ThermoFisher Scientific	Cat# 62-6520, RRID: AB_2533947
Lys63-specific ubiquitin	Millipore	Cat# 05-1308, RRID: AB_1587580
gp120	Abcam	Cat# ab21179, RRID: AB_732949
Polyubiquitin (K63-linkage-specific)	Enzo Life Sciences	Cat# BML-PW0600-0025, RRID: AB_2052278
P24	Abcam	Cat# ab9071, RRID: AB_306981

Data file S1 (Microsoft Excel format). P values in Fig. 4D and fig. S4G.
Data file S2 and **Data file S3**(Microsoft Excel format). All raw data in this manuscript.
